# Combination Analysis of Metatranscriptome and Metagenome Reveal the Composition and Functional Response of Coral Symbionts to Bleaching During an El Niño Event

**DOI:** 10.3389/fmicb.2020.00448

**Published:** 2020-03-20

**Authors:** Fulin Sun, Hongqiang Yang, Guan Wang, Qi Shi

**Affiliations:** ^1^South China Sea Institute of Oceanology, Institute of South China Sea Ecology and Environmental Engineering, Chinese Academy of Sciences, Guangzhou, China; ^2^Key Laboratory of Ocean and Marginal Sea Geology, South China Sea Institute of Oceanology, Chinese Academy of Sciences, Guangzhou, China; ^3^State Key Laboratory of Tropical Oceanography, South China Sea Institute of Oceanology, Chinese Academy of Sciences, Guangzhou, China; ^4^Daya Bay Marine Biology Research Station, Chinese Academy of Sciences, Shenzhen, China; ^5^Nansha Marine Ecological and Environmental Research Station, Chinese Academy of Sciences, Sansha, China; ^6^Southern Marine Science and Engineering Guangdong Laboratory (Guangzhou), Guangzhou, China

**Keywords:** coral symbionts, bleaching, metagenome, metatranscriptome, composition response, functional response

## Abstract

With the abnormal rise in ocean temperatures globally in recent years, coral bleaching is becoming common and serious. However, the response mechanisms and processes of coral symbionts to bleaching are not well understood. In this study, metagenomics and metatranscriptomics were used to explore the composition of coral symbionts and their functions in response to coral bleaching. All four bleaching coral species displayed a significant reduction of the abundance and function of Dinophyceae-like eukaryotes at the DNA and RNA levels. However, different species of bleaching coral have their own characteristic symbiotic components. Bleaching *Acropora tenuis* and *Goniastrea minuta* corals exhibited a very high abundance of prokaryotes and associated gene functions, especially for opportunistic bacteria. In contrast, algae and fungi were identified as the main microbial associate components and had relatively high RNA abundance in bleaching *Pocillopora verrucosa* and *Pocillopora meandrina*. Different coral species, whether unbleached or bleaching, have the same symbiotic taxa that perform the same biological functions *in vivo*. Different stages of bleaching, or transitional states, were identified by different genome content and functional gene abundance among bleaching corals. These stages should be considered in future coral bleaching studies to accurately determine symbiont structure and function. An implicit hypothesis is that there is a causal relationship between the stability of eukaryotic communities and coral bleaching.

## Introduction

El Nino events have a significant impact on global climate, most notably causing warming events, which affect the stability of marine ecosystems (Heron et al., 2016; Hughes et al., 2018a). Widespread bleaching of coral reefs, resulting in high levels of coral mortality due to heat stress (Hoegh-Guldberg, 1999; Heron et al., 2016), is now recognized as a global threat to coral. 2015–2017 were the three warmest years in the instrumental record period since 1880^1^ and record high temperatures triggered a pan-tropical coral bleaching episode (Hughes et al., 2018b). The world’s largest coral reef ecosystem, the Great Barrier Reef, has experienced the highest temperatures ever recorded and lost nearly 30% of coral cover (Hughes et al., 2018a).

Coral is closely related to a complex group of microorganisms, including Symbiodiniaceae, fungi, bacteria, archaea, endolithic algae, and viruses, in a relationship known as coral symbiosis (Reshef et al., 2006). Coral microbes play an important role in nutrient cycling and antimicrobial protection in coral reefs (Ritchie and Smith, 2004; Zhang et al., 2015; Bourne et al., 2016). Therefore, it is important to investigate the effects of bleaching events on the function of coral microbial communities. Recently, metagenomics has been used to investigate the taxonomic diversity and metabolic capabilities of coral-associated microbes under thermal stress or bleaching (Thurber et al., 2009; Littman et al., 2011; Lee et al., 2015; Ziegler et al., 2017). These studies suggested that microbes can undergo major shifts, from symbionts to opportunistic microbes or potential disease-causing bacteria, during heat stress or bleaching (Bourne et al., 2007; Thurber et al., 2009; Littman et al., 2011). In addition to this, the metabolism of the microbial community can shift from autotrophy to heterotrophy (Littman et al., 2011), which involves sulfur and nitrogen metabolism, fatty acid and lipid utilization, and secondary metabolism.

Traditionally, research into coral bleaching has mainly focused on studying photosynthetic symbiotic algae known as Symbiodiniaceae. Physiological damage and expulsion of algal symbionts are thought to be the result of the host immune response triggered by reactive oxygen species produced by coral hosts, algal symbionts, or both (Weis et al., 2008). Research on Symbiodiniaceae has been focused on changes in diversity and density of Symbiodiniaceae (Fine and Loya, 2002; Cunning and Baker, 2012; Howells et al., 2013; Kemp et al., 2014), Photosystem II damage in symbiotic dinoflagellates (Warner et al., 1999), thermal tolerance of Symbiodiniaceae (Ruiz Sebastián et al., 2009; Tong et al., 2017), and functional changes in Symbiodiniaceae (Loram et al., 2007). However, few studies have reported the functional response of Symbiodiniaceae to bleaching (Gierz et al., 2017).

Although previous studies had not focused on eukaryotes, they have hinted that eukaryotes are the most abundant component of coral symbionts (Wegley et al., 2007; Littman et al., 2011). Microeukaryotes have been most widely associated with coral diseases and mortality (Ainsworth et al., 2017). To date, studies on other microeukaryotes associated with coral have mainly focused on several key populations, including fungi, endolithic microalgae and protists. The potential diversity of coral related fungi suggests a broader role beyond pathogenicity (Raghukumar and Ravindran, 2012). Metagenomic analysis has revealed that endolithic algae can play a key role in the microbial community by driving important chemical processes (Littman et al., 2011). When zooxanthellae are absent, other microeukaryotes can provide nutrients that increase coral survival during periods of acute stress (Fine and Loya, 2002; Fine et al., 2006; del Campo et al., 2016). Despite the fact that other eukaryotes are ubiquitous in corals, little is known about their diversity and ecological function during coral bleaching events.

Until now, fundamental gaps have existed in our understanding of the coral microbe and its functional contribution to coral. In 2016, a bleaching event also affected a large area of coral in the South China Sea, where mass coral bleaching had not previously been recorded. In this study, four different coral species were studied to provide an overview of the metagenomic and metatranscriptome response of coral symbionts (prokaryotes, Symbiodiniaceae, other endolithic eukaryotes and the coral itself) to bleaching and to highlight differences in their functional performance. Coral species were collected at the same location to eliminate any potential external environmental influences. In order to study the different components of coral symbionts separately, each symbiotic component was separated based on the NCBI non-redundant (NR) protein database. Combined with KEGG (Kyoto Encyclopedia of Genes and Genomes database) annotation, the corresponding functions of DNA and RNA in different components were explored.

## Materials and Methods

### Sample Collection

**FIGURE 1:**
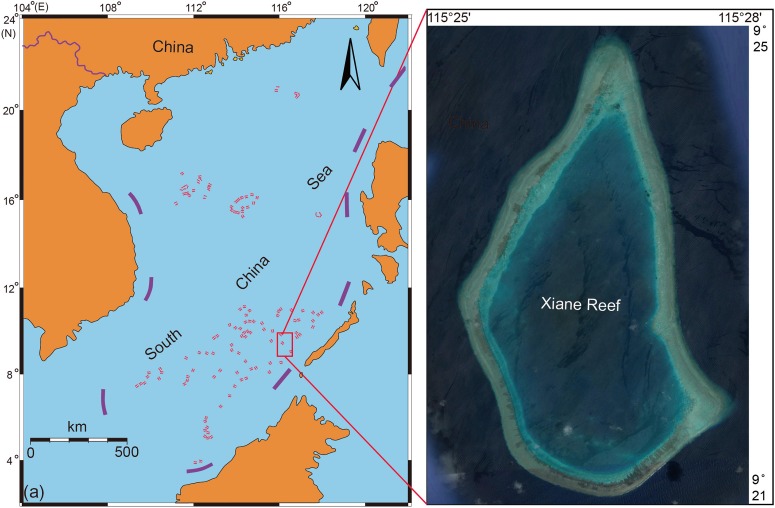
Location map of sampling sites in the South China Sea during an El Nino period.

**FIGURE 2:**
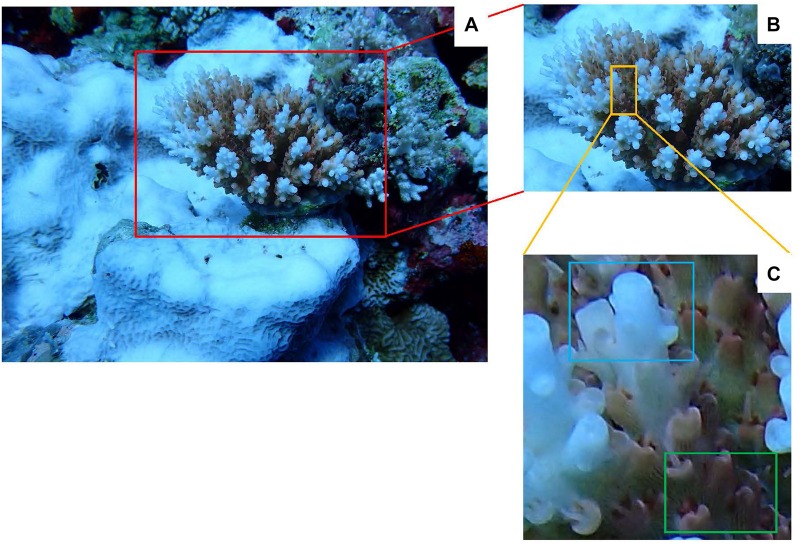
The field sampling process of *Acropora tenuis*. **(A)** *Acropora tenuis* is in bleaching during our sampling time, the lower left part of its growth base is bleached *Favites* sp.; **(B)** This is the red frame part of the **(A)**, *Acropora tenuis* in the bleaching; **(C)** This is the yellow frame of the part **(B)**; the upper blue frame of part **(C)** is the collection part of the bleaching coral sample, and the lower part of the green frame is the collection part of unbleached coral samples.

Coral samples were collected at Xiane reef, Nansha Islands, South China Sea, in June 2016 (Figure 1). Four coral species (unbleached coral and bleached coral collected from the same coral colony), *Acropora tenuis* (AC-H, AC-W), *Goniastrea minuta* (GM-H, GM-W), *Pocillopora verrucosa* (PV-H, PV-W) and *Pocillopora meandrina* (PM-H, PM-W) were sampled. The field sampling process of coral samples was showed in Figure 2. Water temperatures in the sampling area ranged from 30.5 to 31°C. Three replicate samples of unbleached and bleached parts of coral (including tissue, mucus and skeleton) were collected using a hammer and chisel. Once collected, the coral samples (1 cm × 1 cm) were washed with sterile seawater three times to remove any surface attachments. Each sample was divided into two parts and placed in sterile centrifuge tubes with Sample Protector (Takara, Japan) for DNA and RNA extraction. DNA and RNA isolation of replicate samples was mixed and conducted using DNeasy and RNeasy plant mini kit (Qiagen, Germany) following the manufacturer’s instructions. Coral tissues were removed with an airbrush for the identification of each species. All coral samples were identified according to their ecological and morphological characteristics.

### Library Preparation and Metagenome Sequencing

For each coral sample, 200 ng of total DNA was randomly fragmented to <500 bp by sonication (Covaris S220). The fragments were treated with End Prep Enzyme Mix to repair both ends and add a dA-tailing in one reaction, followed by a T-A ligation to add adaptors to both ends. Size selection of Adaptor-ligated DNA was then performed using AxyPrep Mag PCR Clean-up (Axygen), and fragments of ∼410 bp were recovered. Each sample was then amplified by PCR for eight cycles using P5 (5′-AATGATACGGCGACCACCGA) and P7 (5′-CAAGCAGAAGACGGCATACGA) primers. Then libraries were multiplexed and loaded on an Illumina HiSeq instrument (Illumina, United States). Sequencing was carried out using a 2 × 150 paired-end (PE) configuration; image analysis and base calling were conducted by the HiSeq Control Software (HCS) + OLB + GAPipeline-1.6 (Illumina) on the HiSeq instrument.

### Metagenomic Data Analysis

Raw shotgun sequencing reads were trimmed using cutadapt (v1.9.1). Low-quality reads, N-rich reads and adapter-polluted reads were removed. Each samples was assembled *de novo* to obtain separate assemblies. Whole genome *de novo* assemblies were performed using MEGAHIT (v1.13) (Li et al., 2015) with different k-mer (39, 59, 79, and 119). The best assembly result of Scaffold, which has the largest N50, was selected for the gene prediction analysis.

Genes of each sample were predicted using Prodigal (v3.02) (Hyatt et al., 2010). CD-HIT (Fu et al., 2012) was used to cluster genes derived from all samples with a default identity of 0.95 and coverage of 0.9. In order to analyze the relative abundance of unigenes in each sample, paired-end clean reads were mapped to unigenes using SOAPAligner (version 2.2.1) to generate read coverage information for unigenes. Based on the number of mapped reads and the length of gene, the abundance information of each gene was calculated in each sample. The formula is as follows, r represents the number of reads mapped to the genes and L represents gene’s length.

$$G_{k}=\frac{T_{k}}{L_{k}}\cdot \frac{1}{\sum_{i=1}^{n}\frac{r_{i}}{L_{i}}}$$

The unigene sequences were blasted against the constructed microbial database. And the lowest common ancestor was determined using Metagenome Analyzer (MEGAN, v6.4.4). The abundance of a species in one sample equaled the sum of the gene abundance annotated for the species. Diamond (version v0.8.15.77) was used to search the protein sequences of the unigenes with the NR database and KEGG database^2^ with E < 1e-5. The statistical significance threshold of the sequence alignment was set at 1e^–5^ and the sequence alignment length was set as no less than 60% of the reference gene protein length. The matched result with best scores was selected for annotation.

### Library Preparation and Metatranscriptome Sequencing

Total RNA of each sample was quantified and qualified by Agilent 2100 Bioanalyzer (Agilent Technologies, United States) and 1 μg total RNA with a RIN value above 7 was used for following library preparation. The rRNA was depleted from total RNA using Ribo-Zero rRNA Removal Kit (Illumina, United States). The ribosomal depleted mRNA was then fragmented, reverse-transcribed, and used to synthesize first strand cDNA with random primers and Actinomycin *D*. The second-strand cDNA was synthesized using Second Strand Synthesis Enzyme Mix (include dACG-TP/Dutp). The purified double-stranded cDNA by AxyPrep Mag PCR Clean-up (Axygen, China) was then treated with End Prep Enzyme Mix to repair both ends and add a dA-tailing in one reaction, followed by a T-A ligation to add adaptors to both ends. Size selection of Adaptor-ligated DNA was then performed using AxyPrep Mag PCR Clean-up (Axygen, China), and fragments of ∼410 bp were recovered. The dUTP-marked second strand was digested with Uracil-Specific Excision Reagent enzyme (New England Biolabs). Each sample was then amplified by PCR for 14 cycles using P5 and P7 primers. Sequencing was the same as macrogenome operation.

### Data Analysis of Metatranscriptome

Raw shotgun sequencing reads were trimmed using cutadapt (v1.9.1). Low-quality reads, N-rich reads and adapter-polluted reads were removed. The PE reads were assembled using Trinity v2.4.0. Trinity uses the de Bruijn graph strategy to assemble the transcriptome. Open reading frames were identified using the TransDecoder program^3^, with default parameters.

The sequence reads from six samples were individually aligned to the assembled transcriptome, using the aligner bowtie2. The resulting files were then quantified using RSEM (v1.2.4) which default parameters to get gene based raw hit-count data. After adjustment with Benjamini and Hochberg’s approach for controlling the false discovery rate, *P*-value of genes were set < 0.05 to detect differentially expressed genes. The metatranscriptome used FPKM (Fragment Per Kilo bases per Million reads) to calculate gene expression abundance. The FPKM formula is as follows: A_FPKM_ = 10^9^C/NL, where C is the fragment number to compare gene A, N is the total fragment number to compare all genes, L is the base length of gene A. BLASTX was used to search the protein sequences of the predicted genes with the NCBI non-redundant (NR) protein database, CAZy database, eggNOG database and KEGG database with *E* –value cut off of 1e^–5^. Scripts were used in house to enrich the significant differential expression of genes in KEGG pathways.

## Results

### Overview of the Metagenome and Metatranscriptome

**FIGURE 3:**
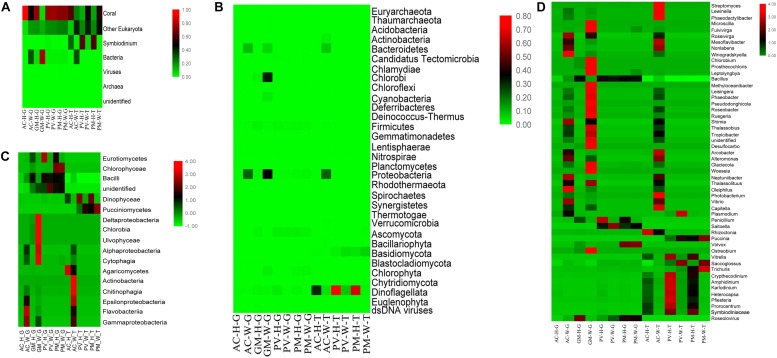
Taxonomic distribution of corals through metagenomic(-G) and metatranscriptomic(-T) profile analysis. **(A)** Coral symbiosis components; **(B)** taxa abundance at the phylum level; **(C)** taxa abundance at the class level; and **(D)** genus abundance with homogenized data.

A summary of the metagenome and metatranscriptome results are shown in Supplementary Tables S1, S2. Figure 3A shows the taxonomy annotations of metagenome and metatranscriptome based on different classification levels. At the DNA level, the abundance of unbleached coral genomes accounted for more than 80% of the total number of sequences, but the DNA content had low abundance in AC-W (48.55%) and GM-W (7.90%) corals. In comparison with unbleached corals, bacterial abundance increased sharply in AC-W (32.53%) and GM-W (78.63%) corals, as did the abundance of eukaryotes in AC-W. At the RNA level, AC-W coral had a reduced abundance of Symbiodiniaceae compared with AC-H coral. In contrast, AC-W coral had a higher abundance of prokaryotes and eukaryotes than AC-H. Unlike AC corals, the abundance of eukaryotes was higher in PV-W and PM-W in comparison to unbleached corals. Symbiodiniaceae abundance was significantly lower in PV-W and PM-W corals compared with PV-H and PM-H corals.

### Composition and Transcriptional Active of Symbiosis Community Between Unbleached Corals and Bleaching Corals

The results showed that there were distinct differences in the composition of symbionts among different corals at RNA and DNA levels (*p* < 0.01, Fisher’s exact test). For AC and GM corals, metagenomic analysis showed that the most obvious response to bleaching was the increase in abundance of bacterial taxa, affiliated with Alphaproteobacteria, Deltaproteobacteria, Epsilonproteobacteria, Gammaproteobacteria (Proteobacteria), Cytophagia (Bacteroidetes) and Chlorobia (Chlorobi), in addition to a significant decrease in Dinophyceae (Dinoflagellata) abundance (Figures 3B,C).

**FIGURE 4:**
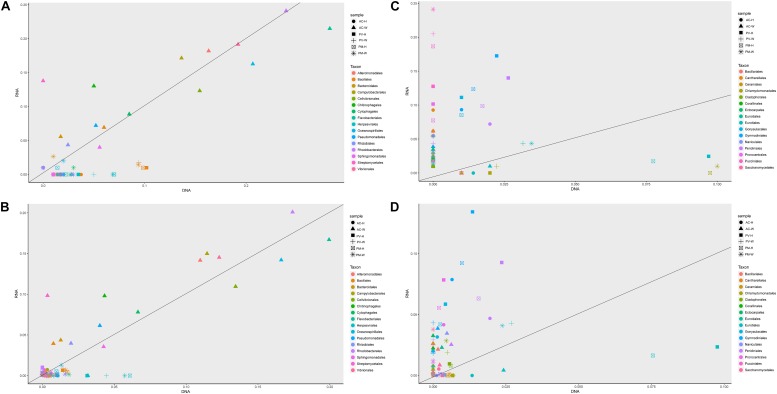
Comparison of 16 major orders and related functions of coral symbionts based on RNA and DNA sequences achieved by metagenomic and metatranscriptomic analysis. **(A)** Prokaryotic orders of coral symbionts; **(B)** function of prokaryotic orders; **(C)** eukaryotic orders of coral symbionts; and **(D)** function of eukaryotic orders.

Furthermore, distinct differences were observed at the class level between unbleached and bleaching corals (Figure 3C). The dominant class of symbionts in PV and PM were Dinophyceae, Eurotiomycetes, Pucciniomycetes (Ascomycota), and Bacilli (Figure 3B). In the case of both unbleached and bleaching PV and PM corals, there was a very low abundance of prokaryotes compared with other symbiont components. Apart from the decreased abundance of Dinophyceae, another feature of bleaching PV and PM corals was the higher abundance of eukaryotes, especially Pucciniomycetes, when compared with unbleached corals. The major genera identified at the RNA and DNA levels varied with samples. Figure 3D shows changes in the structure and abundance of dominant genera between unbleached and bleaching corals. For AC and GM corals, the most obvious response to bleaching in coral symbionts was the shift in abundance of Symbiodiniaceae and bacterial taxa. Bleaching corals exhibited a high diversity and abundance of bacterial taxa at the DNA and RNA level. These taxa included Chlorobiales, Rhodobacterales, Alteromonadales, Oceanospirillales, and Vibrionales. As seen in Figure 4A, more bacterial orders were recorded from AC and GM than PV and PM at the DNA and RNA levels. A higher amount of Campylobacterales, Alteromonadales and Streptomycetale were revealed at the RNA level, in contrast to the results for Rhodobacterales and Vibrionales, which were highly abundant at both the RNA and DNA levels.

For PV and PM corals, the symbiotic component was mainly composed of microeukaryotes, and there was a distinct difference in the abundance and composition for unbleached and bleaching corals (*t*-test, *p* < 0.01). Most notably, Dinophyceae-like genera had a significantly lower abundance in bleaching corals, including *Symbiodiniaceae*, *Crypthecodinium*, *Amphidinium*, *Karlodinium*, *Heterocapsa*, *Pfiesteria*, and *Prorocentrum*. The *Puccinia* genus (Basidiomycota) was dominant at the RNA level, and was more abundant in bleaching than unbleached corals. In unbleached corals, Gymnodiniales, Cantharellales, Peridiniales, Prorocentrales and Gonyaulacales, all of which are affiliated with Dinophyceae, were observed to be more abundant at the RNA and DNA levels in comparison with bleaching corals (Figure 4C).

### Functional Changes of Symbiotic Prokaryotes in Coral

**FIGURE 5:**
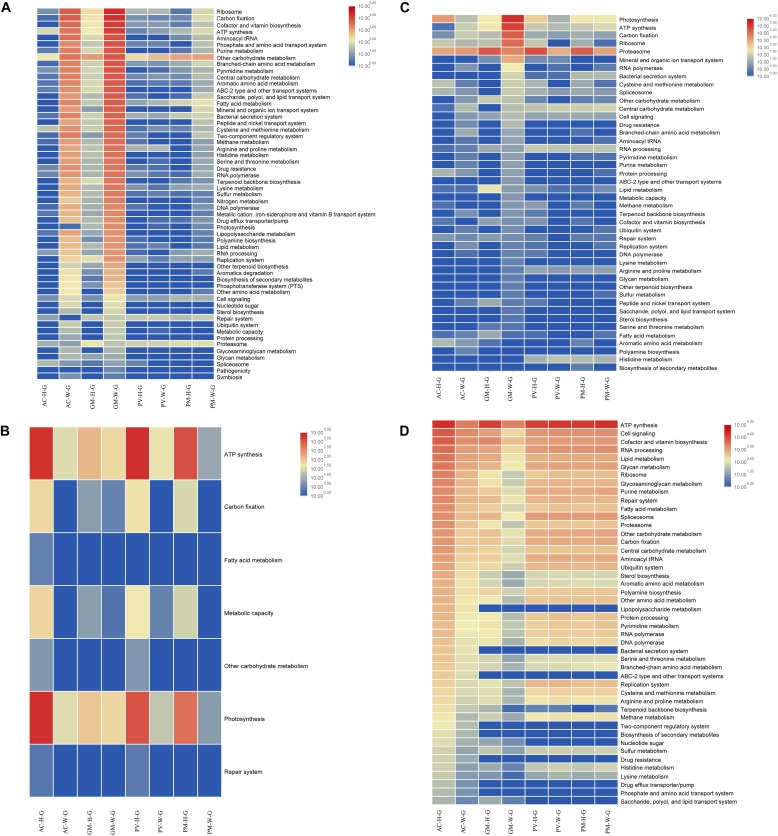
Functional analysis of coral symbiont components through metagenomic analysis. **(A)** prokaryotic function; **(B)** Symbiodinium function; **(C)** eukaryotic function; and **(D)** coral function.

**FIGURE 6:**
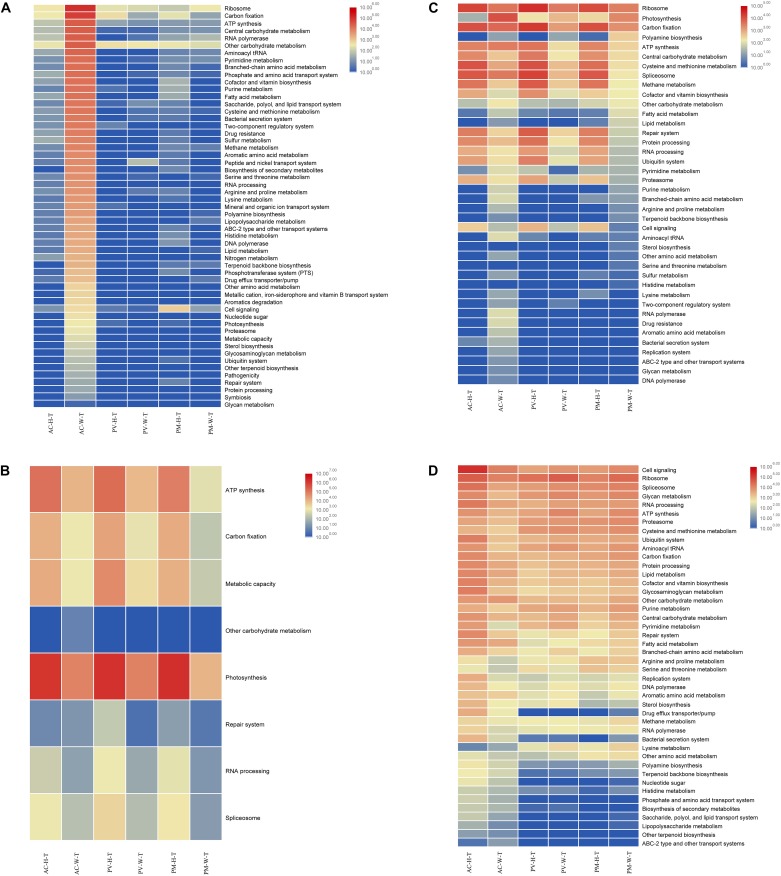
Functional analysis of coral symbiont components through metatranscriptomic analysis. **(A)** Prokaryotic function; **(B)** Symbiodinium function; **(C)** eukaryotic function; and **(D)** coral function.

For AC and GM corals, gene composition and transcriptional abundance increased significantly (*t*-test, *p* < 0.01) in bleaching corals in comparison with unbleached corals (Figures 5A, 6A), including ribosomal, carbon fixation, cofactor and vitamin biosynthesis, and ATP synthesis. Both the metagenomic and metatranscriptome results of the current study indicated that the main contributing prokaryotes for these functions were Proteobacteria, Bacteroidetes, Chlorobi and Actinobacteria (Supplementary Figure S1), and the abundance of bacterial orders was significantly correlated with function, especially at the RNA level (*t*-test, *R*^2^ > 0.8, *p* < 0.01). As seen in Figure 4B, Rhodobacterales, Campylobacterales, Vibrionales and Alteromonadales contributed more to gene function at the RNA level, in contrast to the finding that dominant of Flavobacteriales, Oceanospirillales and Cellvibrionales had a slightly higher contribution to gene function at the DNA level. For PV and PM corals, the bacterial contribution to gene function was very low in both bleaching and unbleached corals (Figure 4B and Supplementary Figure S1).

The abundance of prokaryotes was significantly correlated with functional abundance at the RNA and DNA levels (*t*-test, *R*^2^ > 0.9, *p* < 0.01). For AC and GM corals, carbon fixation pathways, including the reductive citrate cycle (Arnon-Buchanan cycle), the reductive pentose phosphate cycle and the 3-hydroxypropionate bi-cycle, were contributed by Proteobacteria and Bacteroidetes. However, the gene abundance of carbon fixation was very low in unbleached and bleaching PV and PM corals. Dissimilatory nitrate reduction was the main pathway of nitrogen metabolism identified. Proteobacteria, Bacteroidetes, Chlorobi and Tectomicrobia mainly contributed to sulfur metabolism in AC and GM corals. Two of three high abundance sulfur metabolism pathways, assimilatory sulfate reduction and dissimilatory sulfate reduction, were detected in AC and GM corals only. Dominant bacteria, such as Campylobacterales (*Arcobacter* and *Sulfurimonas*) mainly contributed to assimilatory sulfate reduction.

### Functional Changes of Symbiotic Symbiodiniaceae in Coral

The results of metagenomic and metatranscriptomic analysis showed that 24 Symbiodiniaceae species were detected in corals (Supplementary Figure S2). According to the results, all Symbiodiniaceae species decreased in abundance in bleaching corals in comparison with unbleached corals, especially for dominant species *Cladocopium* and *Symbiodinium* sp. ex Scolymia sp.

Of all Symbiodinium types, *Cladocopium* and *Symbiodinium* ex Scolymia sp. were the two genera with the highest abundance of gene composition and transcription. Among the functions performed by Symbiodiniaceae (Figures 5B, 6B), photosynthesis and ATP synthesis were the most important functions. The abundance of Symbiodiniaceae was significantly correlated with functional abundance at the RNA and DNA levels (*t*-test, *R*^2^ > 0.8, *p* < 0.05). In comparison with unbleached corals, Symbiodiniaceae were less abundant in almost all functional genes in bleaching corals. As seen in Supplementary Figure S3, *Cladocopium* and *Symbiodinium* sp. ex Scolymia sp. were the main contributors to photosynthesis. *Cladocopium* and *Symbiodinium minutum* were the main contributors to ATP synthesis. At the RNA level, *Cladocopium* was the main contributor to ATP synthesis in AC corals, while *Cladocopium* and *Symbiodinium minutum* were the main contributors for PV and PM corals. *Cladocopium* was the only performer for RNA processing and Spliceosome at the RNA level. Spliceosome genes in unbleached corals had a higher expression of abundance than bleaching corals, the majority of which belonged to genes involving the heat shock gene (HSPA1_8). There was one carbon fixation pathway (Calvin-Benson cycle) detected in the Symbiodiniaceae. It had a high abundance in all unbleached corals, and exhibited a reduced gene abundance in bleaching corals.

### Functional Changes of Other Eukaryotes (Fungi and Algae) in Corals

The results indicated that most eukaryotes displayed reduced function in bleaching corals compared with unbleached corals. This decline in function resulted in decreased proteasome function, carbon fixation, ATP synthesis and central carbohydrate metabolism. However, photosynthetic activity was found to increase in all bleaching corals, in particular AC coral (Figures 5C, 6C). As seen in Supplementary Figure S4, the main contributors to function are the dominant Dinophyceae (including Gymnodiniales, Peridiniales, Prorocentrales, and Gonyaulacales) and Eurotiales. A higher number of Gymnodiniales, Peridiniales, Prorocentrales and Gonyaulacales contributed to function at the RNA level, in contrast to the DNA level, where Eurotiales mostly contributed to the function (Figure 4D). It was unexpectedly found that Dinophyceae were the main contributors to all functions except photosynthesis (Supplementary Figure S4). Interestingly, there was an obvious increase in the abundance of genes involved in photosynthesis in bleaching coral, mainly attributed to Bacillariophyceae, Florideophyceae, and Trebouxiophyceae. The Calvin cycle is the main pathway for carbon fixation of these eukaryotes. There was a lower abundance of genes involved in carbon fixation in bleaching corals compared with unbleached corals. Dinophyceae were the main contributor to Spliceosomes, and the abundance of the heat shock 70 gene was significantly reduced in bleaching corals compared with unbleached corals.

### Functional Comparison of Unbleached and Bleaching Corals

Almost all of the functions of AC-W coral were reduced compared with unbleached corals, in particular cell signaling, ribosomal activity, spliceosome activity, glycan metabolism, RNA processing and ATP synthesis (Figures 5D, 6D). In contrast to AC coral, almost all functions of PV-W and PM-W were increased in comparison with unbleached corals (Figure 6D).

## Discussion

### Bleaching Significantly Altered Coral Bacterial Community and Increased Its Functional Gene Abundance

An obvious response to bleaching displayed by coral symbionts was the shift in abundance of bacteria. Bleaching corals exhibited a higher diversity and abundance of bacterial taxa at the DNA and RNA level than unbleached corals, indicating that bacteria were easily affected by coral bleaching. Many studies have shown that potentially opportunistic microbes can sharply increase in abundance and become dominant in bleaching corals (Mouchka et al., 2010; Kumar et al., 2016), causing the coral to move toward an unstable state (Thurber et al., 2009; Littman et al., 2011; Zaneveld et al., 2016; McDevitt-Irwin et al., 2017), even inducing coral disease (Rosenberg et al., 2007; Kimes et al., 2012; Maynard et al., 2015). Coral bleaching can alter the chemical composition of coral mucus, increase organic matter and mucus production (Niggl et al., 2009), which induces a shift in the coral-associated microbial community (Raina et al., 2013; Lee et al., 2016). The results of the current study indicated that these opportunistic microbes were highly abundant in bleaching coral, and may have resulted in an elevation of bacterial-organic matter coupling (Figure 4A).

Almost all functional genes of prokaryotes were improved in bleaching AC and GM corals. Previous studies demonstrated that the metabolism of the microbial community could shift from autotrophy to heterotrophy under stress, resulting in an increase in the abundance of microbial genes involved in sulfur and nitrogen metabolism, and secondary metabolism (Thurber et al., 2009; Littman et al., 2011). As the microbial community shifts from autotrophy to heterotrophy, bacterial consumption of organic matter becomes greatly enhanced, and the contribution of fixed N_2_ and photosynthesis for nitrogen and carbon budgets became less obvious. High abundance sulfur metabolism pathways (assimilatory sulfate reduction and dissimilatory sulfate reduction) were detected in bleaching AC and GM corals, increasing the possibility of producing sulfide. High abundance of these heterotrophic bacteria could deplete nutrients in coral, and deteriorate the microenvironment, ultimately making coral bleaching irreversible.

### Bleaching Significantly Reduced Functional Gene Abundance of Symbiotic Symbiodiniaceae

According to the results of the current study, the abundance of all Symbiodiniaceae obviously decreased in all bleaching corals in comparison with unbleached corals, indicating that the nutrition supplied to coral by Symbiodiniaceae decreased. Exocytosis or *in situ* symbiotic degradation during bleaching seemed to be less invasive and cost effective than host cell degradation (Oakley and Davy, 2018). This study identified that the four coral species could simultaneously host a very high diversity of genotypic Symbiodiniaceae phylotypes, more than described by other studies (Toller et al., 2001; Kemp et al., 2008). *Cladocopium* is often regarded as a sensitive species to temperature or bleaching (Kemp et al., 2014), and is dominant in scleractinian corals in the South China Sea. *Cladocopium* were the main contributors to photosynthesis and ATP synthesis in coral.

This result provided direct evidence that bleaching may have important effects on photosynthesis via the inhibition of the Calvin cycle, limiting carbon fixation in Symbiodiniaceae, as described in previous studies (Jones et al., 1998; Wooldridge, 2016). It had been found that carbon fixation via the Calvin cycle is sensitive to heat stress (Murata et al., 2007). Previous studies reported that the maximum quantum yield of photosystem II was significantly lower and highly variable in bleaching corals in comparison with healthy corals (Kemp et al., 2014). This occurred with a corresponding loss of electron flow supporting carbon fixation (Slavov et al., 2016). ATP synthesis was essential for repair in photosystem II (Murata et al., 2007), therefore the repair rate that this study suggested was decreased through the inhibition of ATP synthesis in bleaching coral. In addition, a low abundance of the heat shock gene indicated that bleaching inhibited the activity of heat-inducible genes and heat acclimation of Symbiodiniaceae (Rosic et al., 2011) resulting in the reduced ability of Symbiodiniaceae to resist thermal stresses.

### Importance of Other Eukaryotes (Fungi and Algae) in Bleaching Corals

Another finding of the current study was that, when corals are bleaching, they not only expel Symbiodiniaceae, but all of the identified Dinophyceae genera, which indicates that these algae have the same response mechanism to coral bleaching. Other eukaryotic algae also showed distinct changes in bleaching corals compared to unbleached corals. It has been reported that when Symbiodiniaceae are absent, these algae could provide an alternative source of photoassimilates, and provide nutrients that increase coral survival during stress (Fine et al., 2006; del Campo et al., 2016). The current study also suggested that fungi displayed high abundance of RNA in bleaching PV and PM corals, and bleaching stimulated fungal growth. Some studies have suggested that fungi were also thought to be opportunistic pathogens, and their abundance depended on coral health (Yarden et al., 2007; Toledo-Hernández et al., 2008).

The functional genes of other eukaryote algae increased in abundance during bleaching, indicating that the photosystems of other eukaryotes (such as Bacillariophyta, Chlorophyta) used a different mechanism and had higher levels of thermal tolerance compared to Dinophyceae algae. It was also thought that other endosymbiotic algae benefit the host coral during periods of stress (Gutiérrez-Isaza et al., 2015; del Campo et al., 2016). This may be because during bleaching, the shading effect of Symbiodiniaceae was lost, allowing increased light to penetrate the coral skeleton, possibly resulting in increased photosynthetic activity of these algae. Given the ability of microalgae to adapt rapidly to heat stress, these populations might become important as “secondary” symbionts (Fine and Loya, 2002; Fine et al., 2005), and continue to provide nutrients for coral through photosynthesis.

### Functional Gene Change in Coral at Different Stages of Coral Bleaching

The results of the current study indicated that the coral symbionts under investigation were at different stages of bleaching. Almost all of the functions of bleaching AC and GM coral were reduced compared with unbleached corals (Figures 5D, 6D), suggesting that the nucleic acid of bleaching corals was being degraded and was in an apoptotic state. A reduction in mRNA abundance of cytochrome c and ATP synthase, which were central components of the respiratory electron transport chain (Dunn et al., 2012), inhibited the ability of the host to survive or recover from thermal stress (Oakley and Davy, 2018). This suggested that bleaching AC and GM corals lost most of their physiological metabolic activity and function.

However, almost all functions of bleaching PV and PM were increased in comparison with unbleached corals. This indicated that coral was in a temporary stage of transformation from an unbleached to a seriously bleached state. At this stage, corals exhibit symptoms of bleaching as they have expelled most symbiotic algae; however, the symbiotic structure in bleaching corals has certain similarities in composition to unbleached corals. The remaining Symbiodiniaceae and other algae play an important role in bleaching coral, providing nutrition to the host and maintaining coral activities under stress.

## Conclusion

**FIGURE 7:**
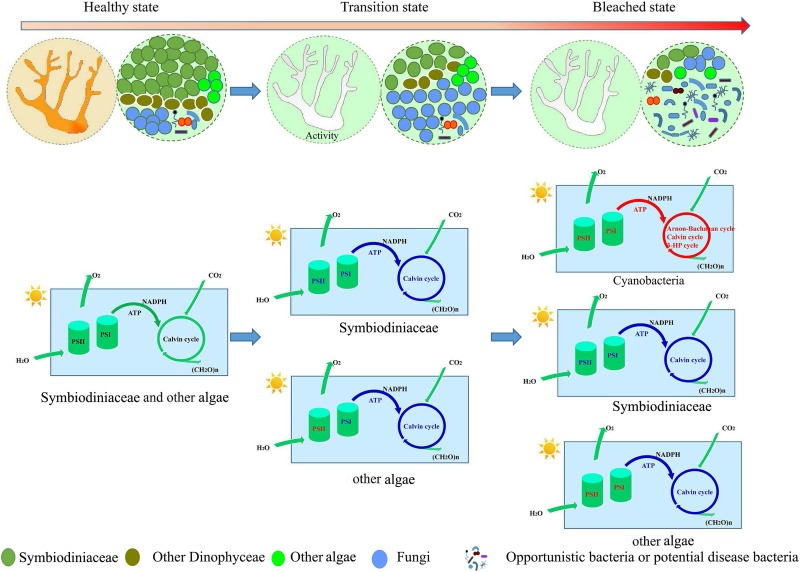
A hypothetical conceptual map summarizing the shift of coral symbionts during bleaching, illustrating the functional changes in photosynthesis and carbon fixation in different coral symbiont components. Blue represent down regulation of function; Red represent up regulation of function.

For both unbleached and bleaching coral, it was found that different coral species have common symbiotic taxa that perform biological functions *in vivo*. Overall, different coral species were found to have common characteristics when bleached: a decreased abundance of Symbiodiniaceae and associated function and the exclusion of Dinophyceae-like eukaryotes. Furthermore, this study might reflect the different stages of the coral bleaching process. In the early stages of coral bleaching, algae such as Symbiodiniaceae and other Dinophyceae were expelled from corals. If the coral microbe could maintain a level of stability similar to that of an unbleached coral, the coral itself could retain its functional activity. Otherwise, the coral itself gradually decreased in activity due to the lack of nutrients usually provided by algae. Opportunistic bacteria then multiply in large numbers, and result in the deterioration of the coral microenvironment (Figure 7). In fact, symbionts are more often in a transient state of disturbance than one of stability. This study suggested that when coral microbes are studied during bleaching, the activity of the coral itself should also be considered.

## Data Availability Statement

The raw sequence data was deposited in the NCBI Sequence Read Archive under the BioProject PRJNA525179.

## Author Contributions

FS and HY conceived the research and performed all the experiments. FS, HY, GW, and QS performed data analysis and revised the manuscript. FS wrote the manuscript. HY and GW contributed the materials and identified coral species.

## Conflict of Interest

The authors declare that the research was conducted in the absence of any commercial or financial relationships that could be construed as a potential conflict of interest.

## Footnotes

1 https://www.ncdc.noaa.gov/sotc/global/201710

2 https://www.kegg.jp/

3 https://github.com/TransDecoder/TransDecoder/wiki

## Supplementary Material

FIGURE S1 Main contributing prokaryote for functions through metagenomic and metatranscriptome analysis. **(A)** DNA level; **(B)** RNA level.

FIGURE S2 Abundance of Symbiodinium species in different corals through metagenomic and metatranscriptome sequencing.

FIGURE S3 Main contributing Symbiodinium for functions through metagenomic and metatranscriptome analysis. **(A)** DNA level; **(B)** RNA level.

FIGURE S4 Main contributing other eukaryote for functions through metagenomic and metatranscriptome analysis. **(A)** DNA level; **(B)** RNA level.

TABLE S1 Information summary of the metagenome data.

TABLE S2 Information summary of the metatranscriptome data.
