# Supplementary figures and images for: Combination Analysis of Metatranscriptome and Metagenome Reveal the Composition and Functional Response of Coral Symbionts to Bleaching During an El Niño Event
Source: Front Microbiol. 2020 Mar 20;11:448. doi: 10.3389/fmicb.2020.00448 (PMC7104784; doi:10.3389/fmicb.2020.00448)

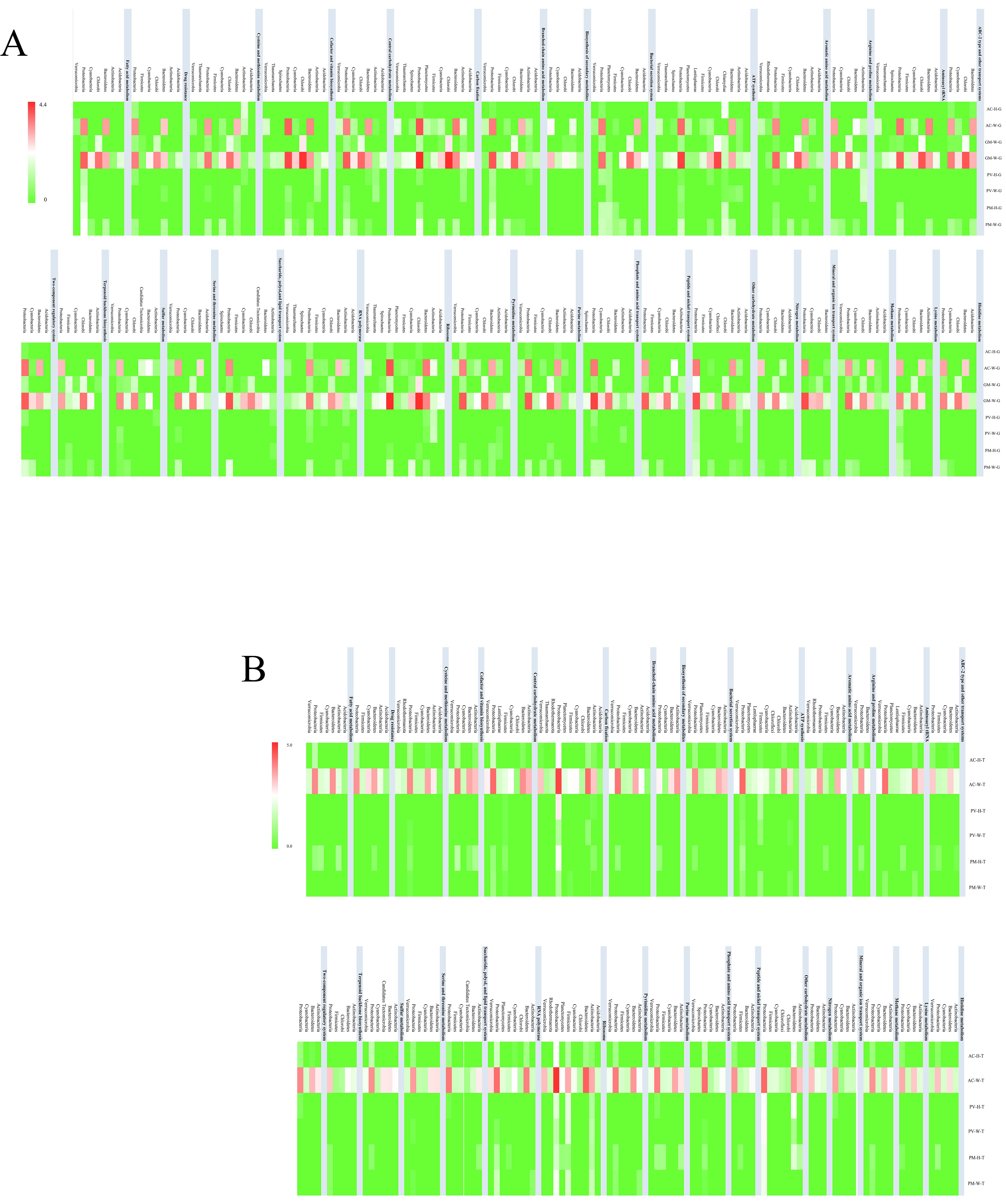

Supplement: FIGURE S1 — Main contributing prokaryote for functions through metagenomic and metatranscriptome analysis. (A) DNA level; (B) RNA level. [file Image_1.TIF]

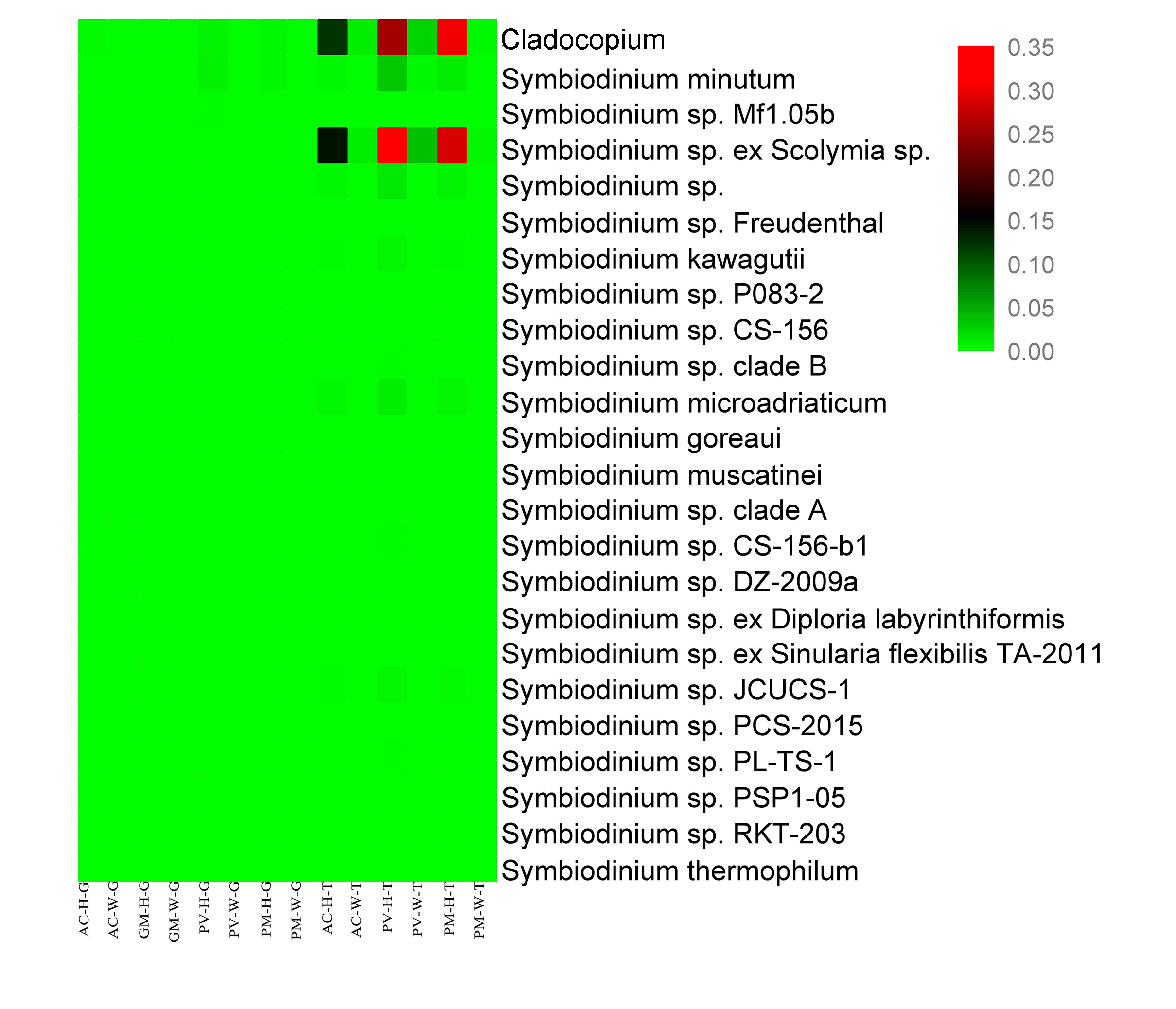

Supplement: FIGURE S2 — Abundance of Symbiodinium species in different corals through metagenomic and metatranscriptome sequencing. [file Image_2.TIF]

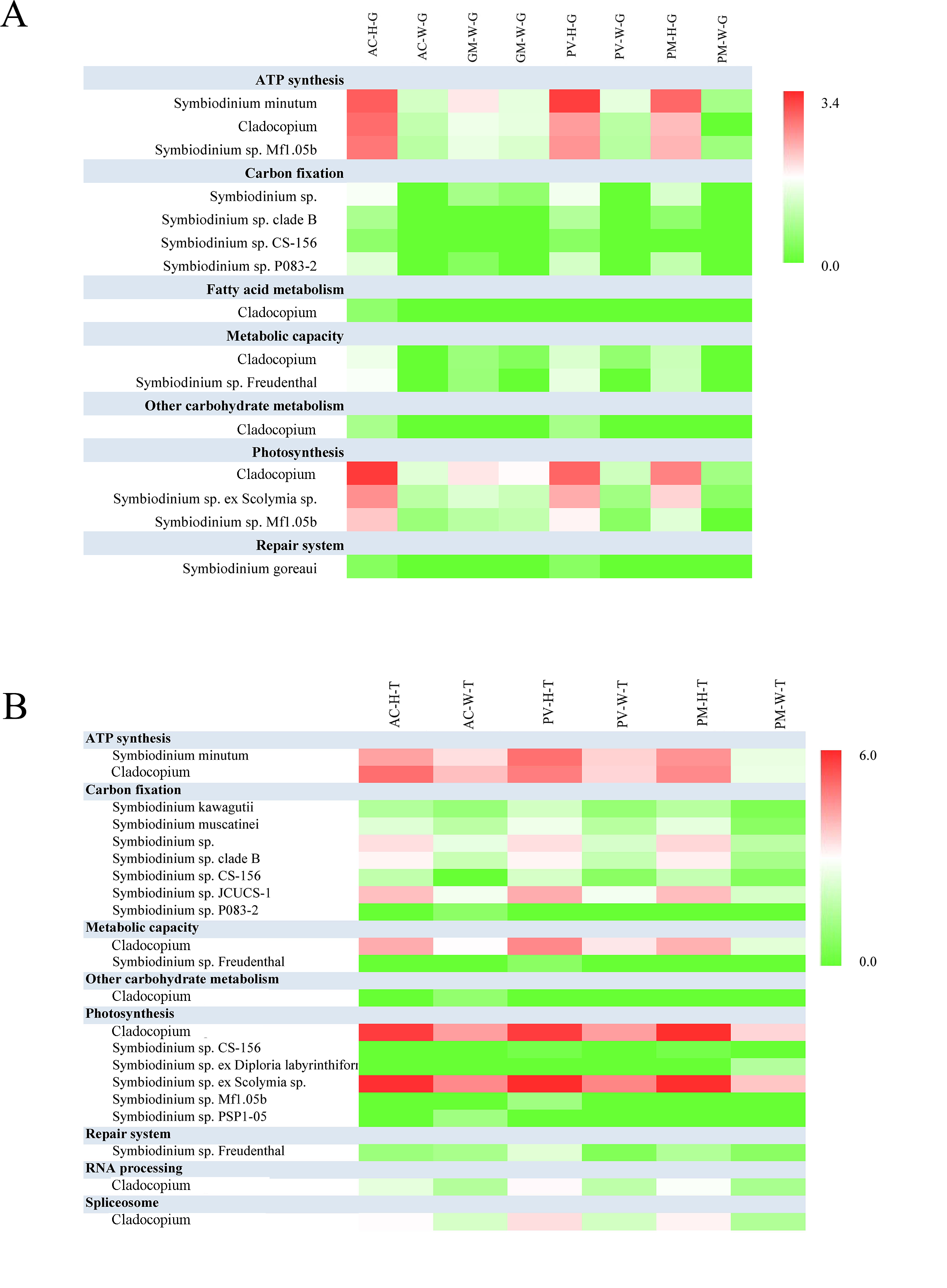

Supplement: FIGURE S3 — Main contributing Symbiodinium for functions through metagenomic and metatranscriptome analysis. (A) DNA level; (B) RNA level. [file Image_3.TIF]

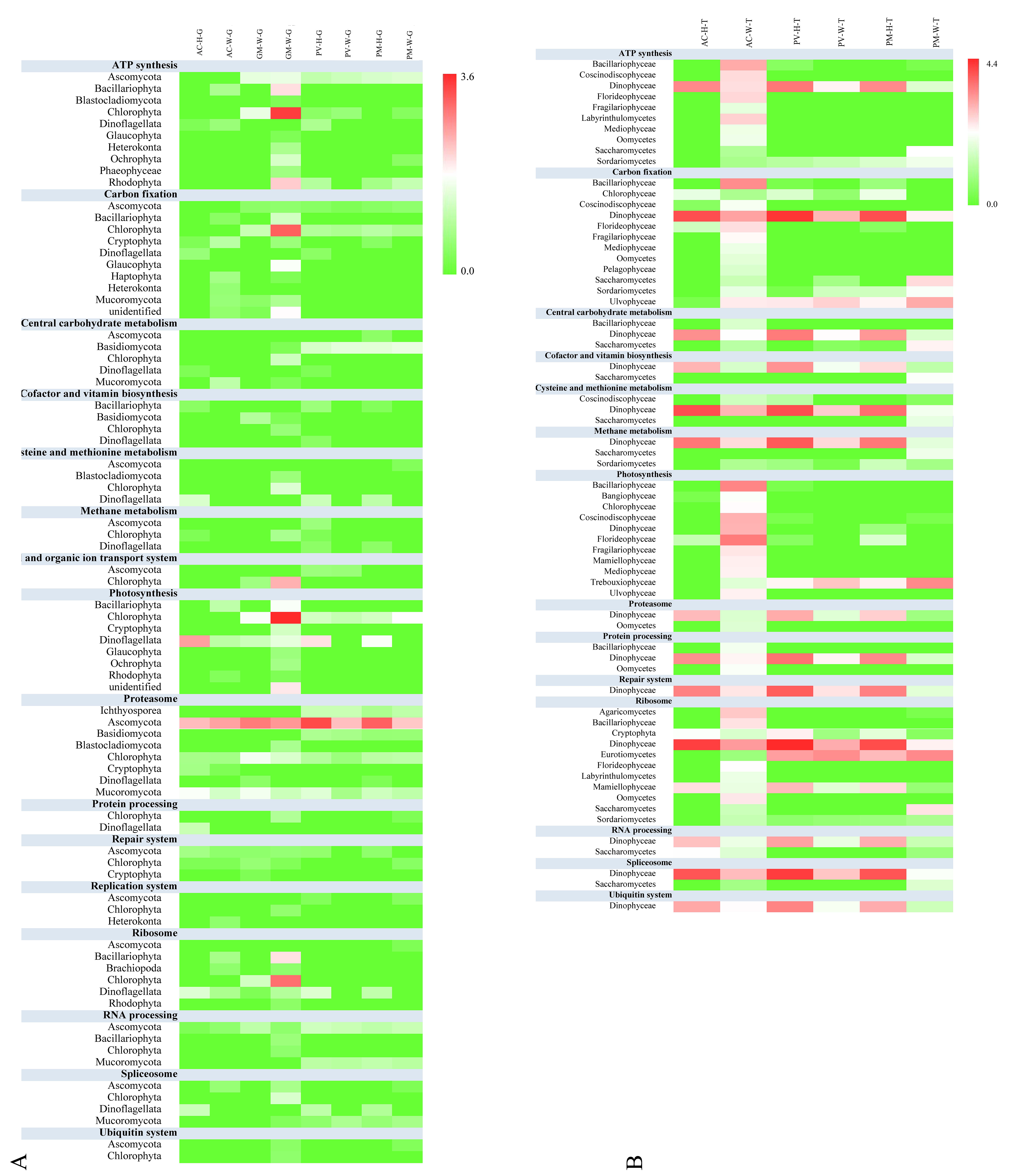

Supplement: FIGURE S4 — Main contributing other eukaryote for functions through metagenomic and metatranscriptome analysis. (A) DNA level; (B) RNA level. [file Image_4.TIF]
