# Supplementary material for: Combination Analysis of Metatranscriptome and Metagenome Reveal the Composition and Functional Response of Coral Symbionts to Bleaching During an El Niño Event
Source: Front Microbiol. 2020 Mar 20;11:448. doi: 10.3389/fmicb.2020.00448 (PMC7104784; doi:10.3389/fmicb.2020.00448)
Supplement: TABLE S1 — Information summary of the metagenome data. [file Table_1.docx]

Table S1 Information summary of the metagenome data

| Sample | AC-H-G | AC-W-G | GM-H-G | GM-W-G | PV-H-G | PV-W-G | PM-H-G | PM-W-G |
| --- | --- | --- | --- | --- | --- | --- | --- | --- |
| Total number of raw reads | 149562424 | 112324378 | 152218056 | 158460188 | 150245510 | 153133484 | 153234056 | 168487544 |
| Clean Reads | 146946080 | 110145216 | 150159556 | 155007942 | 147154840 | 150623252 | 150976618 | 165867734 |
| Ratio of Reads (%) | 98.25 | 98.06 | 98.65 | 97.82 | 97.94 | 98.36 | 98.53 | 98.45 |
| Total assembled contigs | 741052 | 1011188 | 770552 | 1612598 | 654413 | 355266 | 488155 | 386370 |
| Average contig length (bp) | 975.9 | 1128.24 | 1072.96 | 946.35 | 958.16 | 1252.04 | 1056.52 | 1195.32 |
| Average (G + C)s% content of assembled contigs (%) | 41.22 | 41.57 | 39.88 | 45.42 | 40.18 | 37.92 | 38.98 | 38.22 |
| Total number of genes encoding in the contigs | 841398 | 1355619 | 817098 | 1963728 | 697012 | 403551 | 528219 | 432178 |
