# Supplementary material for: Combination Analysis of Metatranscriptome and Metagenome Reveal the Composition and Functional Response of Coral Symbionts to Bleaching During an El Niño Event
Source: Front Microbiol. 2020 Mar 20;11:448. doi: 10.3389/fmicb.2020.00448 (PMC7104784; doi:10.3389/fmicb.2020.00448)
Supplement: TABLE S2 — Information summary of the metatranscriptome data. [file Table_2.docx]

Table S2 Information summary of the metatranscriptome data

| Sample | AC-H-T | AC-W-T | PV-H-T | PV-W-T | PM-H-T | PM-W-T |
| --- | --- | --- | --- | --- | --- | --- |
| Total number of raw reads | 158887076 | 171242874 | 174244492 | 165831380 | 226287608 | 176603442 |
| Clean Reads | 156621060 | 168114536 | 172590682 | 163662908 | 223151970 | 173752768 |
| Ratio of Reads (%) | 98.57 | 98.17 | 99.05 | 98.69 | 98.61 | 98.39 |
| Total assembled contigs | 674156 | 1127523 | 587046 | 654031 | 920069 | 699075 |
| Average contig length (bp) | 361.64 | 260.92 | 374.41 | 329.94 | 314.04 | 326.64 |
| Average (G + C)s% content of assembled contigs (%) | 42.80 | 43.56 | 43.55 | 39.00 | 41.47 | 38.69 |
| Total number of genes encoding in the contigs | 2395366 | | | | | |
